# Supplementary material for: Demethylation of methylguanidine by a stepwise dioxygenase and lyase reaction
Source: Nat Commun. 2025 Oct 8;16:8957. doi: 10.1038/s41467-025-64776-2 (PMC12508155; doi:10.1038/s41467-025-64776-2)
Supplement: Supplementary file 1 — Supplementary Information [file 41467_2025_64776_MOESM1_ESM.pdf]

Supplementary Information for

Demethylation of methylguanidine by a stepwise dioxygenase and lyase reaction

M. Sinn[1], D. Funck[1], F. Gamer[1], C. Blumenthal[1], C. Kramp[1] and J. S. Hartig[1,2]

[1] Department of Chemistry, University of Konstanz, Germany

[2] Konstanz Research School Chemical Biology (KoRS-CB), University of Konstanz, Germany

Content:

Supplementary Fig. 1: Whole radiograph of the Gd-I in-line probing assay
Supplementary Fig. 2: In-line probing assay of Gd-II Rsw with N,N-dimethylguanidine
Supplementary Fig. 3: SDS-PAGE of purified MgdL
Supplementary Fig. 4: Chai-1 model and conservation analysis of MgdL
Supplementary Fig. 5: MgdL variants
Supplementary Fig. 6: LC-MS analysis of the demethylation of N,N-dimethylguanidine
Supplementary Fig. 7: Methylguanidine and guanidine as N-source
Supplementary Fig. 8: SIM traces of compounds detected in the MgdH reactions
Supplementary Fig. 9: Standard curve of the Nash reaction
Supplementary Table 1: Compounds tested in the MgdH substrate screen
Supplementary Table 2: Sequences (5’-3’) of oligonucleotides used for in-line probing assay

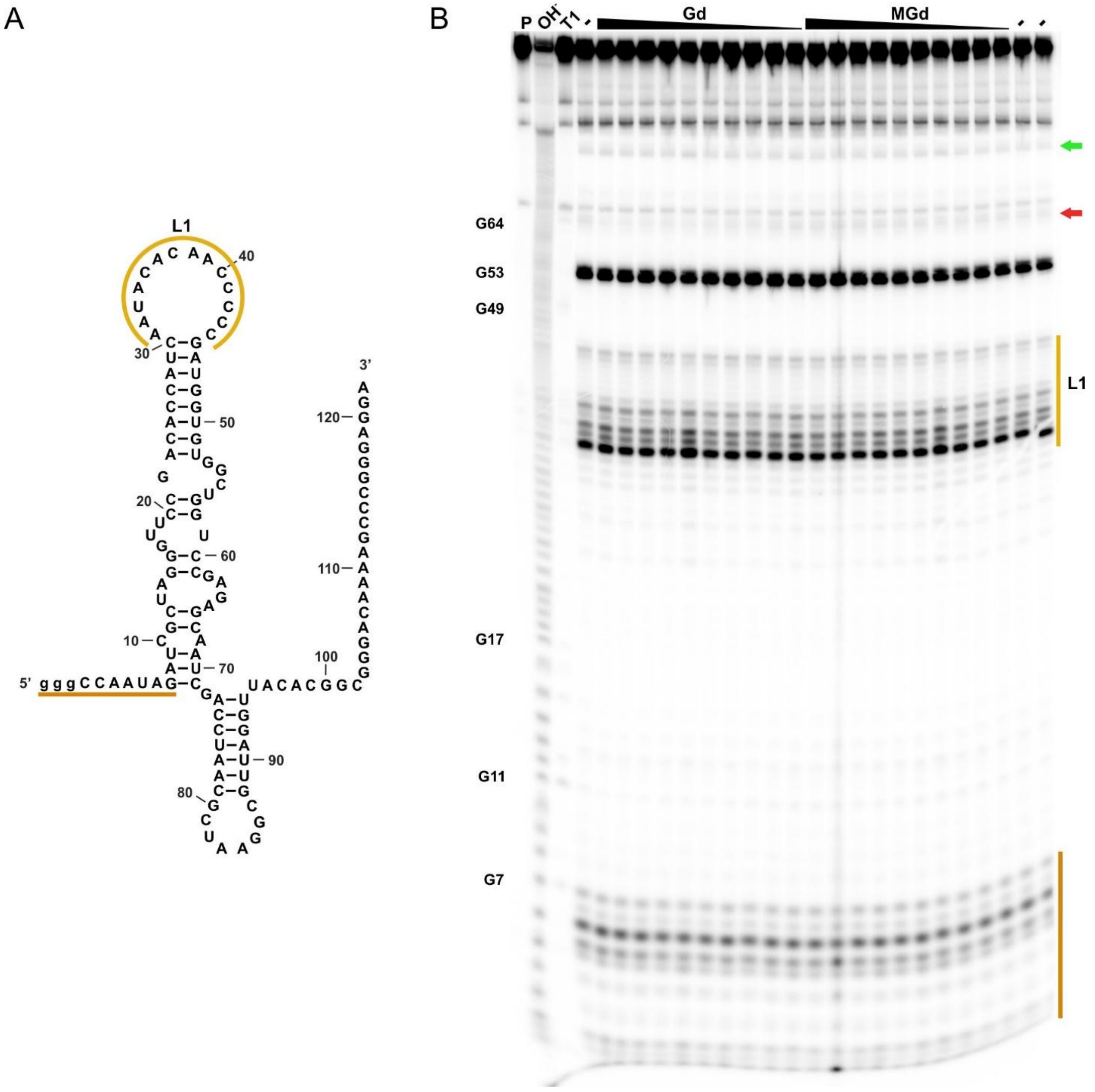

Supplementary Fig. 1: In-Line-probing of the Gd-I riboswitch. A Secondary structure of the Gd-I riboswitch preceding the *gca* operon in *V. boliviensis*. Secondary structure is based on R2DT 2.0 and Reiss *et al.* [1,2] Binding of guanidine involves H-bonding with G64 and G120 as well as cation-π stacking with G100 and G116. [2] **B** Radiograph of a Gd-I riboswitch in-line probing assay with guanidine (Gd) and methylguanidine (MGd) (full picture of figure 2A). RNA labelled with ^{32}P at the 3′ end was incubated under slightly alkaline conditions to promote intrinsic cleavage of the phosphate backbone in the presence of decreasing concentrations of Gd or MGd (1 mM - 5 µM). Guanidine (Gd) but not methylguanidine (MGd) induced regions/bands of reduced (red) cleavage. Single-stranded regions of the Gd-I riboswitch highlighted in A are annotated accordingly. Precursor RNA (P) and reactions without ligand (-) were included on the gel as controls, as well as an alkaline digest (OH^-) of the RNA and a digest with RNAse T1, which cleaves specifically at guanosines (T1) for nucleotide assignment.

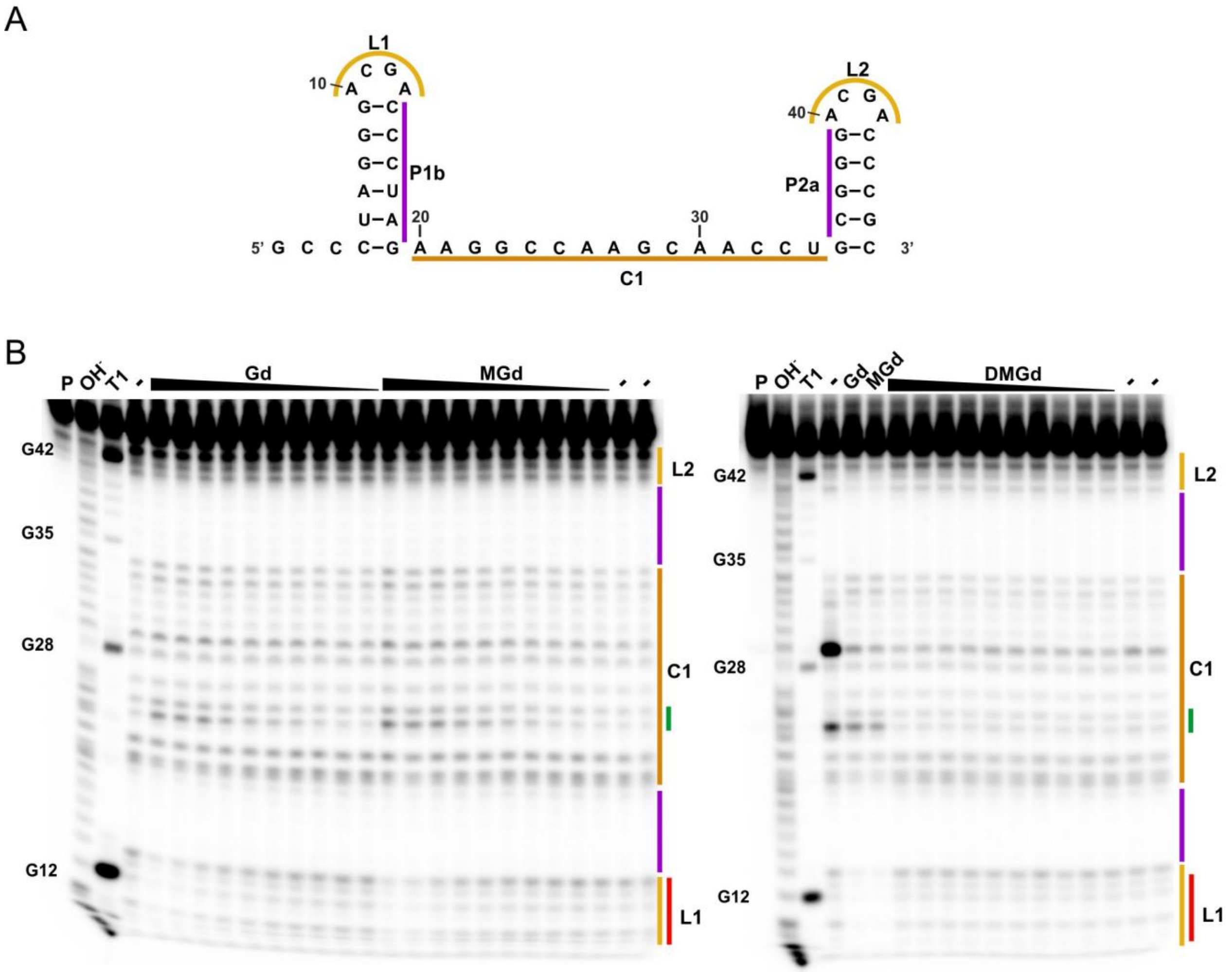

Supplementary Fig. 2: In-Line-probing of the Gd-II riboswitch. A Secondary structure of the Gd-II riboswitch directly upstream of *gca* in *V. boliviensis*. Guanidine binding occurs in loop 1 (L1) and loop 2 (L2) [3,4]. **B** Radiograph of a Gd-II riboswitch in-line probing assay with guanidine (Gd), methylguanidine (MGd) (full picture of figure 2B) and N,N-dimethylguanidine (DMGd). RNA labelled with ^{32}P at the 3′ end was incubated under slightly alkaline conditions to promote intrinsic cleavage of the phosphate backbone in the presence of decreasing concentrations of Gd, MGd or DMGd (1 mM- 5 µM) and 1 mM Gd and MGd on the right gel. Gd and MGd induced regions/bands of reduced (red) and enhanced (green) cleavage, whereas no change in cleavage pattern was observed for DMGd. The loop and stem regions of the Gd-II riboswitch are highlighted according to the annotation in A. Precursor RNA (P) and reactions without ligand (-) were included on the gel as controls, as well as an alkaline digest (OH^-) of the RNA and a digest with RNAse T, which cleaves specifically at guanosines (T1) for nucleotide assignment. Note that in one of the control reactions (-) on the right gel unspecific cleavage was observed.

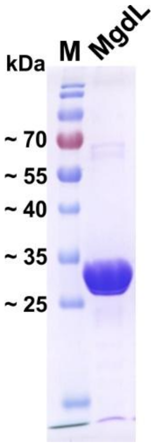

Supplementary Fig. 3: Purified recombinant MgdL. Representative denaturing polyacrylamide gel of 6xHis-tagged MgdL (Uniprot Acc. No. A0A265DXW6) purified by nickel affinity chromatography. The predicted molecular mass of MgdL including the 6xHis tag is 30.1 kDa.

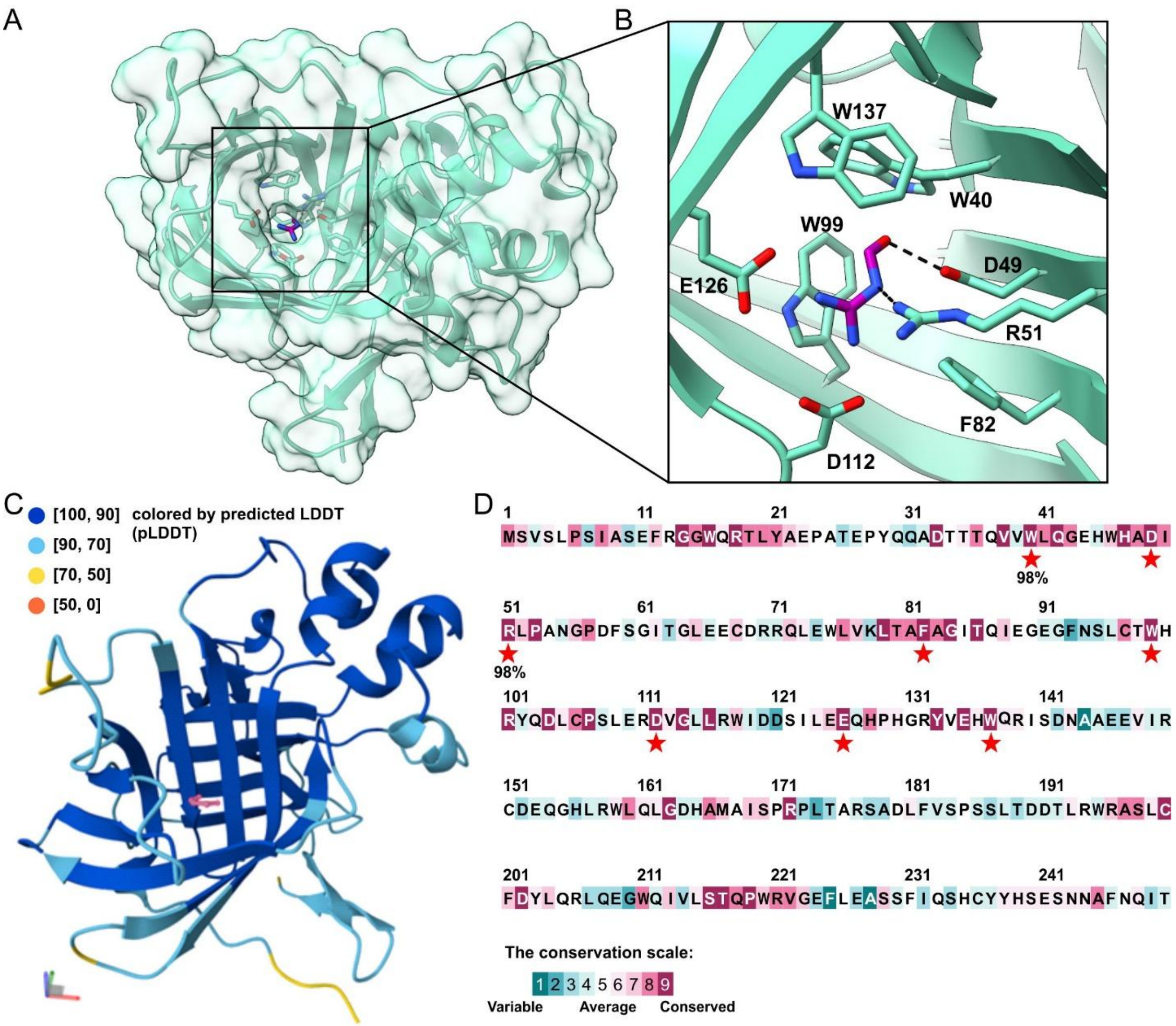

Supplementary Fig. 4: Three-dimensional model of MgdL with bound substrate. A The Chai-1 webserver[5] was used to predict the atomic structure of MgdL and the binding of N-(hydroxymethyl)-guanidine within the structure. The predicted structure of MgdL comprises a β-barrel core flanked by a second β-sheet, which is connected to the β-barrel by short α-helices. **B** Close-up of the β-barrel core with the putative catalytic center and the substrate N-(hydroxymethyl)guanidine. Side chains of amino acids within 5 Å of the substrate are shown as stick models. E126 and D112 could form hydrogen bonds with the guanidine moiety of the substrate. D49 and R51 could be involved in an acid-base catalytic mechanism for cleavage of the connective carbon-nitrogen bond to release formaldehyde (see main Figure 4). **C** Side view of the predicted structure of MgdL colored according to the local confidence score calculated by the predicted local distance difference test (pLDDT).[5] The substrate N-(hydroxymethyl)-guanidine is highlighted in purple. **D** MgdL sequence colored according to the conservation score calculated by the Consurf server.[6] Homologues were retrieved from the Uniprot90 database using HMMER with a minimum sequence identity of 30% and an HMMER e-value of ≤ 0.001. 150 sequences, randomly sampling the total list of 216 homologues, were used for the calculation. Residues of the predicted binding pocket are marked with red asterisks. The conservation of these residues is 100%, if not stated otherwise.

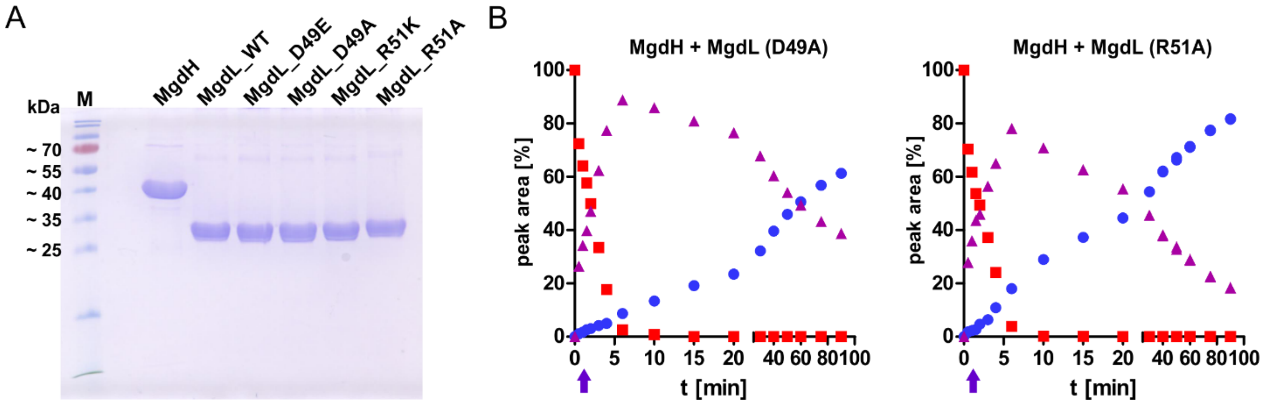

Supplementary Fig. 5: MgdL variants. A Purified recombinant MgdH, wiltype MgdL (MgdL_WT) and MgdL variants used in the activity assays to test the activity of the mutated versions. In the variants, D49 and R51 were mutated to alanine (D49A and R51A) or to more similar glutamate (D49E) and lysine (R51L), respectively. **B** Activity assays with MgdH, MgdL and methylguanidine were set up as described in Figure 4. Substrate disappearance and product formation was monitored by LC-MS. MgdL variants D49A and R51K were added after 1.5 min as indicated by the purple arrows. Data represent single data points. Consistent results were obtained with independent enzyme preparations.

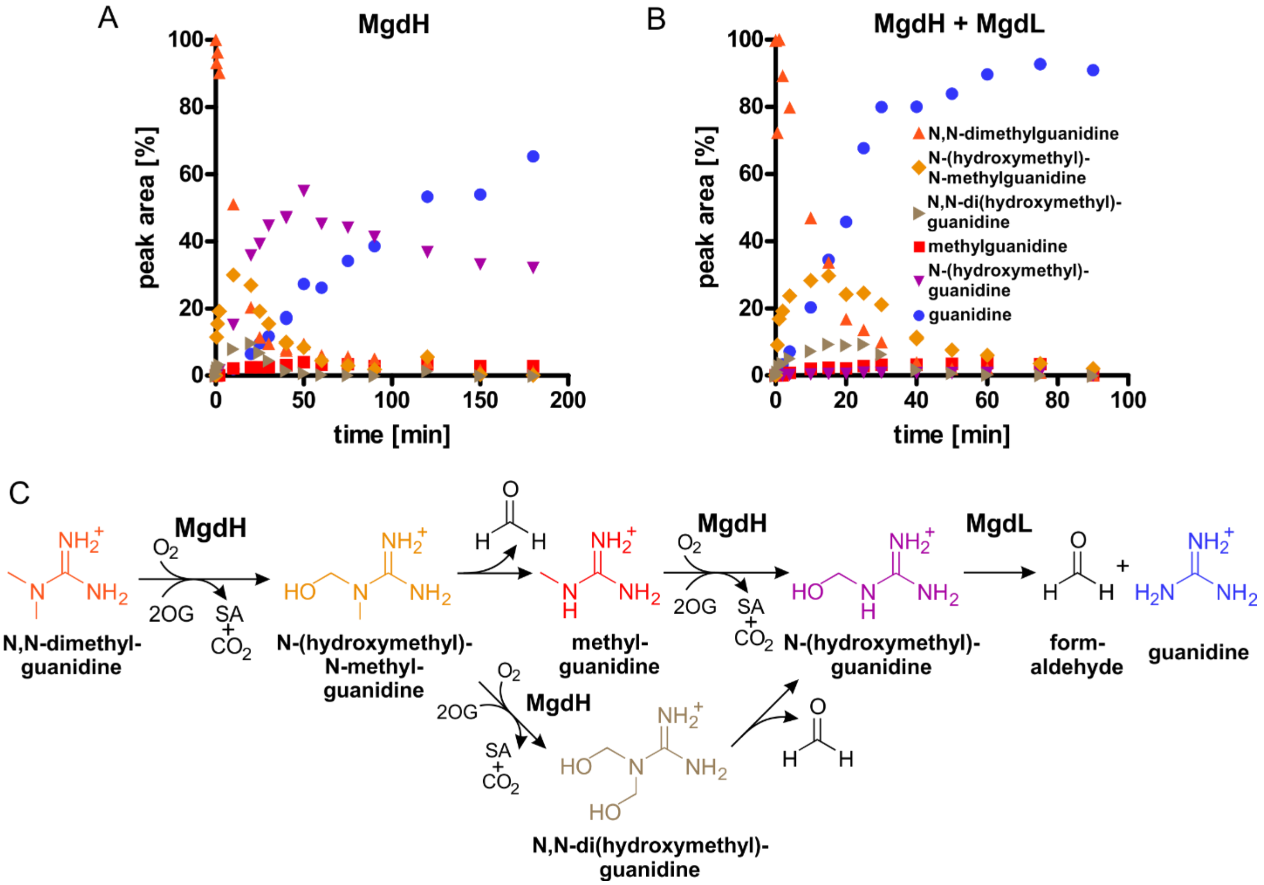

Supplementary Fig. 6: LC-MS analysis of the demethylation of dimethylguanidine. A 0.5 mM N,N-dimethylguanidine were incubated with 5 mM 2-OG and 10 µg/mL MgdH. Aliquots of the reaction were removed at different time points and subjected directly to LC-MS. Consumption of N,N-dimethylguanidine (orange triangles) and generation of hydroxymethylated intermediates, methylguanidine (red squares) and guanidine (blue dots) were monitored by measuring the respective *m/z* ratios in positive single ion mode. N-(hydroxymethyl)guanidine (purple triangles) slowly decayed to guanidine and was still observed after 3 h. In contrast, first intermediates N-hydroxymethyl-N-methylguanidine and N-di(hydroxymethyl)-guanidine were formed and decayed within 1 h. Note that no substantial levels of methylguanidine were built up. **B** The reaction was set up as described in A, with the addition of 20 µg/mL MgdL. In contrast, to the reaction without MgdL no substantial levels of N-(hydroxymethyl)guanidine were observed, whereas the formation and degradation of the other hydroxymethylated intermediates was unaffected. The results indicate that MgdL exhibits selectivity towards N-(hydroxymethyl)guanidine and does not accept the hydroxylated N,N-dimethylguanidine species as substrates. **C** Scheme of the demethylation reactions of N,N-dimethylguanidine with hydroxymethylated intermediates to guanidine as monitored in A and B. Coloring of the chemical structures corresponds to that of the data in A and B. Data represent single data points. Consistent results were obtained with independent enzyme preparations.

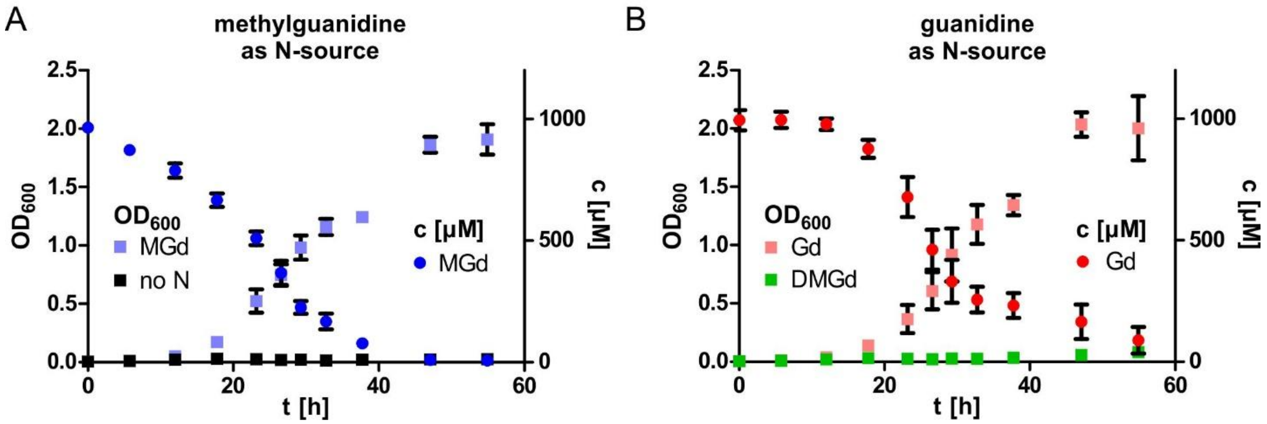

Supplementary Fig. 7: Methylguanidine and guanidine as N-source. *V. boliviensis* was grown in minimal medium with glucose as carbon and energy source and guanidine or methylguanidine as the sole nitrogen source. Growth of *V. boliviensis* was monitored by measuring the OD600 (squares). Simultaneously, substrate consumption was assessed by measuring the methylguanidine and guanidine concentration in the respective cultures by LC-MS (dots). **A** The medium was supplemented with 1 mM methylguanidine as nitrogen source (blue). Medium without N-source was inoculated with *V. boliviensis* as control (black). **B** The medium was supplemented with 1 mM guanidine (red) or 0.25 mM N,N-dimethylguanidine (green). A lower concentration of N,N-dimethylguanidine was used to assess if the larger amount of produced formaldehyde led to the growth deficiency observed (Figure 5). OD_{600} was measured in cuvettes. Error bars indicate standard deviation of three independent cultures. (n=3; error, s.d.)

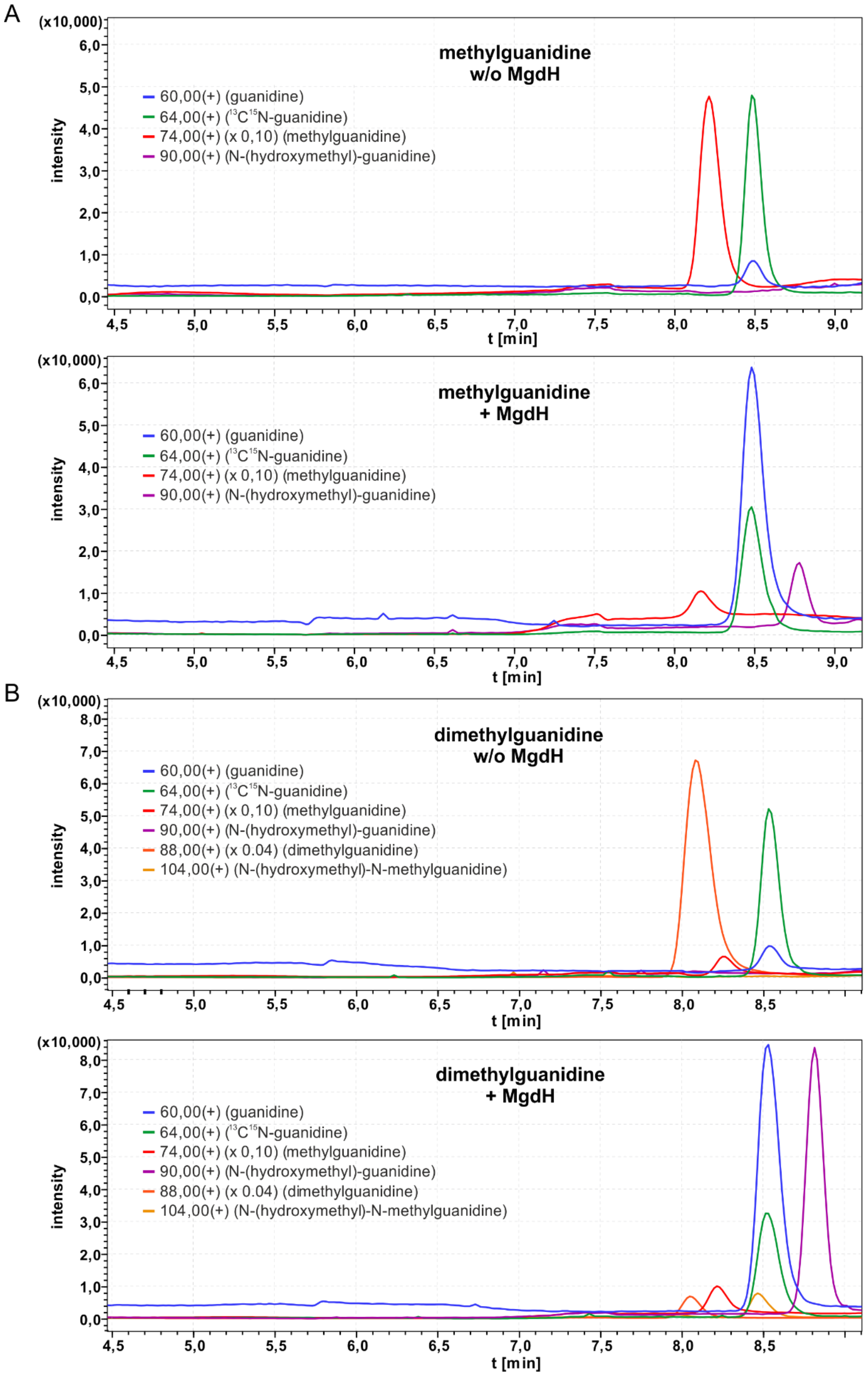

Supplementary Fig. 8: SIM traces of compounds detected in the MgdH reactions. Representative LC-MS chromatograms for the substrates and their products in the MgdH hydroxylation reactions. Assays with methylguanidine (**A**) or dimethylguanidine (**B**) were set up without or with MgdH. Isotopically labelled guanidine ($^{13}C^{15}N$-guanidine) was used as an internal standard.

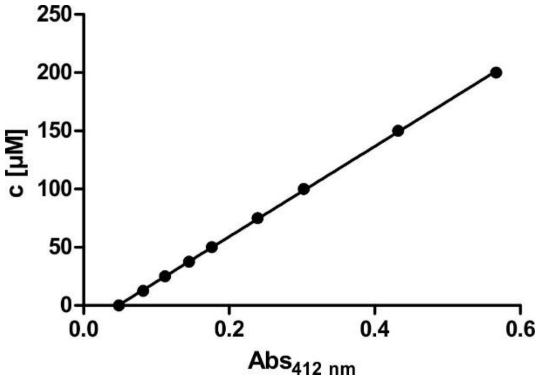

Supplementary Fig. 9: Standard curve of formaldehyde detection. Known concentrations of a freshly prepared formaldehyde dilution series were incubated as described in the methods with Nash reagent and the absorbance at 412 nm was plotted against the formaldehyde concentration. In the tested concentration range (10 to 200 µM), there was a linear correlation between Abs_{412} and formaldehyde concentration (R^2 = 0.99).

Supplementary Table 1: Compounds tested in the MgdH substrate and co-substrate screen. Substrates are highlighted in green.

methylguanidine	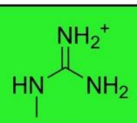	guanidine	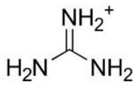
dimethylguanidine	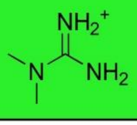	arginine	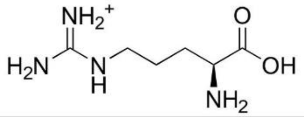
methylamine	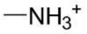	homoarginine	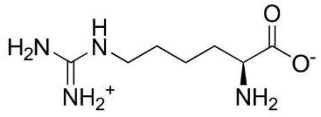
creatine	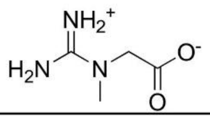	guanidinobutyrate	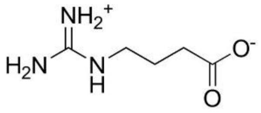
creatinine	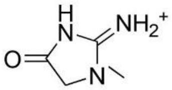	guanidinopropionate	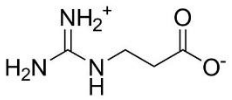
assymetric dimethylarginine	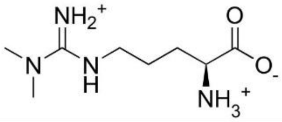	guanidinoacetate	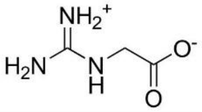
symetric dimethylarginine	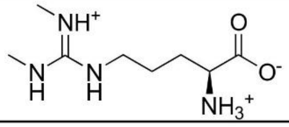	taurocyamine	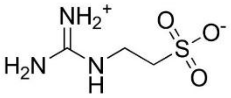
mono-methylarginine	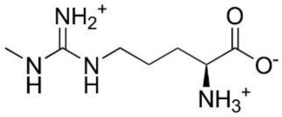	aminoguanidine	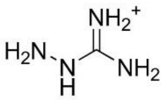
metformin	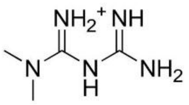	cyanoguanidine	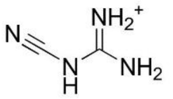

2-oxoglutarate	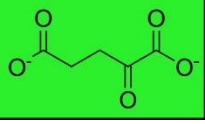	2-oxoadipate	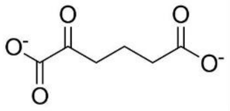	2-oxosuccinate	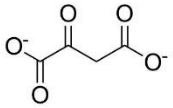

Supplementary Table 2: Sequences (5'-3') of oligonucleotides used for in-line probing assay. In the Gd-I-fwd oligo, three guanosines (highlighted by lower-case) were introduced after the T7 promoter to improve *in-vitro* transcription.

Gd-I fwd	TAATACGACTCACTATAgggCCAATAGATCGCTAGGGTTCCGACACCATCAATA CACAACCCCCGATGGTGTGGCTGGTCCG
Gd-I rev	TCCTCCCGGGCTTTTGTCCCGCCGTGTAACCTAACGCCTTAGCGTTAGGTCGAT TGCTCTCGGACCAGCCACACC
Gd-II fwd	TAATACGACTCACTATAGCCCTAGGGACGACCCTAGAAGGCCAAG
Gd-II rev	GCGGGTCGTCCCGCAGGTTGCTTGGCCTTCTAGGGTCG

Supplementary References

1 McCann, H. *et al.* R2DT: a comprehensive platform for visualizing RNA secondary structure. *Nucleic Acids Res.* **53**, gkaf032 (2025). https://doi.org/10.1093/nar/gkaf032

2 Reiss, C. W., Xiong, Y. & Strobel, S. A. Structural Basis for Ligand Binding to the Guanidine-I Riboswitch. *Structure* **25**, 195-202 (2017). https://doi.org/10.1016/j.str.2016.11.020

3 Sherlock, M. E., Malkowski, S. N. & Breaker, R. R. Biochemical Validation of a Second Guanidine Riboswitch Class in Bacteria. *Biochemistry* **56**, 352-358 (2017). https://doi.org/10.1021/acs.biochem.6b01270

4 Huang, L., Wang, J. & Lilley, D. M. J. The Structure of the Guanidine-II Riboswitch. *Cell Chem Biol* **24**, 695-702 e692 (2017). https://doi.org/10.1016/j.chembiol.2017.05.014

5 discovery, C. *et al.* Chai-1: Decoding the molecular interactions of life. *bioRxiv*, 2024.2010.2010.615955 (2024). https://doi.org/10.1101/2024.10.10.615955

6 Ashkenazy, H. *et al.* ConSurf 2016: an improved methodology to estimate and visualize evolutionary conservation in macromolecules. *Nucleic Acids Res.* **44**, W344-350 (2016). https://doi.org/10.1093/nar/gkw408
