# Supplementary material for: Demethylation of methylguanidine by a stepwise dioxygenase and lyase reaction
Source: Nat Commun. 2025 Oct 8;16:8957. doi: 10.1038/s41467-025-64776-2 (PMC12508155; doi:10.1038/s41467-025-64776-2)
Supplement: Supplementary file 2 — Description of Additional Supplementary Files [file 41467_2025_64776_MOESM2_ESM.pdf]

Description of Additional Supplementary Files

File Name: Supplementary Data 1

Description: Multiple sequence alignment of MgdL homologues

File Name: Supplementary Data 2

Description: Semi-quantitative proteome data of *V. boliviensis* grown on different nitrogen sources
